# Multi-Modal Profiling Reveals Contrasting Immunomodulatory Effects of Recreational Marijuana Used Alone or with Tobacco in Youth with HIV

**DOI:** 10.3390/cells14161267

**Published:** 2025-08-16

**Authors:** Samiksha A. Borkar, Guglielmo M. Venturi, Kai-Fen Chang, Jingwen Gu, Li Yin, Jerry Shen, Bernard M. Fischer, Upasana Nepal, Isaac D. Raplee, Julie J. Kim-Chang, David M. Murdoch, Sharon L. Nichols, Lisa B. Hightow-Weidman, Charurut Somboonwit, John W. Sleasman, Maureen M. Goodenow

**Affiliations:** 1 Molecular HIV and Host Interactions Section, National Institute of Allergy and Infectious Diseases, National Institutes of Health, Bethesda, MD 20892, USA; kai-fen.chang@nih.gov (K.-F.C.); li.yin@nih.gov (L.Y.); jshen100@umd.edu (J.S.); nepal.upasana@nih.gov (U.N.); isaac.raplee@nih.gov (I.D.R.); maureen.goodenow@nih.gov (M.M.G.); 2 Division of Allergy and Immunology, Department of Pediatrics, Duke University School of Medicine, Durham, NC 27710, USA; guglielmo.venturi@duke.edu (G.M.V.); bernie.fischer@duke.edu (B.M.F.); julie.kim.chang@duke.edu (J.J.K.-C.); john.sleasman@duke.edu (J.W.S.); 3 Bioinformatics and Computational Biosciences Branch, Office of Cyber Infrastructure and Computational Biology, National Institute of Allergy and Infectious Diseases, National Institutes of Health, Bethesda, MD 20892, USA; jingwen.gu@nih.gov; 4 Division of Pulmonary, Allergy, and Critical Care Medicine, Department of Medicine, Duke University School of Medicine, Durham, NC 27710, USA; david.murdoch@duke.edu; 5 Department of Neurosciences, University of California, San Diego, CA 92093, USA; slnichols@health.ucsd.edu; 6 Institute on Digital Health and Innovation, College of Nursing, Florida State University, Tallahassee, FL 32306, USA; lhightowweidman@fsu.edu; 7 Division of Infectious Diseases, Department of Medicine, Morsani College of Medicine, University of South Florida, Tampa, FL 33620, USA; charurut@usf.edu

**Keywords:** youth with HIV, youth without HIV, marijuana, tobacco, bioprofiles, biomarkers, flow cytometry, transcriptome, immunomodulatory, comorbidities

## Abstract

The evolving legal landscape has increased marijuana accessibility across the United States, including for medical use to manage clinical symptoms among people with HIV. The effects of marijuana use remain understudied in youth with HIV (YWH), who face lifelong antiretroviral therapy (ART) and an elevated risk of developing comorbidities. This study applied a multi-modal approach, including plasma biomarker analysis, peripheral blood cell phenotyping, and transcriptome profiling, to examine the effects of recreational marijuana alone, tobacco alone, or marijuana combined with tobacco in virally suppressed YWH (≤50 RNA copies/mL) on ART compared to youth without HIV and YWH who used no substance. Marijuana use alone was associated with elevated IL-10 levels and normalization of pro-inflammatory genes and pathways, suggesting an immunomodulatory effect. Conversely, tobacco use alone or combined with marijuana was linked to increased IL-1β levels and heightened pro-inflammatory responses, including upregulation of genes involved in inflammasome activation. This study is the first to demonstrate *GPR15* upregulation and potential marijuana-associated epigenetic modulation in HIV-suppressed youth. The findings identify potential markers for early detection of inflammation-related comorbidities in YWH, particularly among those exposed to tobacco and underscore the need for targeted profiling to guide personalized monitoring and early substance use intervention strategies for YWH.

## 1. Introduction

Marijuana (cannabis) use policy continues to evolve across the United States (U.S.). As of 2024, 40 states have legalized medical marijuana, while 24 states, along with the District of Columbia, have legalized recreational use [1,2]. This shifting legal landscape continues to broaden access to marijuana across diverse populations, including people with HIV (PWH) [3]. While HIV/AIDS remains a qualifying condition for medical marijuana use in all states, the short- and long-term effects of marijuana on health outcomes, particularly in the context of chronic diseases like HIV, remain largely unknown.

Marijuana is one of the most commonly used substances among PWH, for recreational and medical use, with reported estimates ranging from 23% to 56%, compared to 13% in the general U.S. population [4,5]. Use of marijuana alone or in combination with tobacco is especially prevalent among youth, including youth with HIV (YWH) aged 18 to 25 [6,7,8,9,10]. Marijuana contains multiple cannabinoids, including Δ^9^-tetrahydrocannabinol (THC) and cannabidiol (CBD), which exert their effects primarily through the activation of cannabinoid (CB) receptors. These G protein-coupled receptors form the endocannabinoid system and have immunomodulatory properties that reduce chronic inflammation [11,12], a key driver of comorbidities and long-term health complications in PWH [13,14,15]. CB receptors are expressed on multiple cell types involved in immunity and inflammation, including T cells and monocytes [16,17,18,19]. Both in vivo and in vitro studies have shown that cannabinoids reduce levels of pro-inflammatory cytokines such as IL-8, IL-6, and CXCL10 and attenuate macrophage activation [20,21,22,23,24,25,26,27].

However, the overall impact of marijuana use in PWH remains complex and, at times, contradictory. While some studies associate marijuana use with beneficial clinical implications in PWH, such as neurocognitive protection, lower levels of inflammatory markers in blood and spinal fluid, reduced T cell activation, impaired viral entry, lower HIV viral load, and higher CD4 counts [21,28,29,30,31,32,33,34], other studies have identified adverse associations. These include decreased adherence to ART in older PWH [5,35], impaired cognitive function associated with heavy cannabis use in PWH [32,36,37], and an increased cardiovascular risk [38]. HIV-associated immune dysfunction and inflammation are predominantly driven by ongoing viral replication. Yet, even with optimal suppression of viral replication by combinational antiretroviral therapy (ART), ongoing inflammation, driven primarily by macrophage activation, continues and remains a challenge [39,40,41,42].

This study focuses on a cohort of YWH with ART-suppressed viral replication who use recreational marijuana, tobacco, or marijuana in combination with tobacco. Two comparison groups included YWH who used no substance and youth without HIV (YWOH) who used no substances. This study population is ideal to examine the effects of substance use as YWH have fewer HIV-associated comorbidities, more intact immunity, and face decades of ART [43,44]. Furthermore, in 2023, nearly 19% of the approximately 31,000 newly diagnosed HIV infections in the U.S. occurred among youth aged 18 to 25 years [45,46], making this group a key target population to reduce the national HIV epidemic. While the detrimental effects of tobacco alone are well documented [47,48], the effect of marijuana alone or the combined impact of marijuana and tobacco use in the context of HIV-associated inflammation remains poorly studied. This is particularly important given the high rates of marijuana and co-use among YWH. To address this knowledge gap, the current study employed a multi-modal approach, including plasma immune biomarker analysis, lymphocyte subset analysis by flow cytometry, and transcriptome profiling of peripheral blood cells.

## 2. Materials and Methods

### 2.1. Study Participants and Enrollment

The current cross-sectional study involved the use of de-identified clinical data and cryopreserved blood and plasma samples from 138 participants, including 101 YWH and 37 YWOH, enrolled in several multi-center clinical trials throughout the U.S. The YWH participants were enrolled under two protocols: (1) the Adolescent Medicine Trials Network (ATN) for HIV/AIDS Intervention Protocol 071/101, Assessment of Inflammatory Markers Associated with Neurocognitive Impairment in HIV-infected Adolescents, a 3-year longitudinal study conducted at 22 metropolitan sites throughout the U.S. and Puerto Rico (ClinicalTrials.gov Identifier NCT00683579; and (2) the Consequences of Marijuana Use on Inflammatory Pathways in HIV-Infected Youth, with participants recruited from the University of North Carolina at Chapel Hill and University of South Florida (ClinicalTrials.gov Identifier NCT03927053). A control group of YWOH participants was enrolled at the University of South Florida, under the protocol Substance Use and Immunity Function in HIV+ Adolescents by Systems Biology (OHSRP 18-NIAID-00677). Across all protocols, participants completed harmonized questionnaires and underwent standardized clinical and demographic data collection to ensure consistency in data capture across study cohorts. All participants provided written informed consent to enroll in protocols approved by their respective Institutional Review Boards (IRBs). Biological specimens along with clinical and demographic information were stored in a biorepository located at Duke University according to the protocol “Consequences of Marijuana Use on Inflammatory Pathways in HIV-Infected Youth”, reviewed and approved by the IRBs of the National Institute of Allergy and Infectious Diseases, National Institutes of Health, and Duke University School of Medicine. Full details of these study participants have previously been described [8,24,39,40,41,42,49,50].

### 2.2. Study Design

Samples for the current study were selected from study participants with HIV (YWH, *n* = 101), aged 18 to <29 years, who acquired HIV infection through sexual transmission, initiated combination ART regimens early in the disease, before CD4 T cell decline (>350 cells/µL), remained stable for over 12 months on their initial ART, and maintained <50 HIV RNA copies/mL for over six months prior to and at the time of blood collection. Exclusion criteria included a history of AIDS-defining illnesses, pregnancy, significant cognitive impairment or other chronic medical conditions, or acute illnesses. The co-enrolled YWOH (*n* = 37) used no substances, had no acute illnesses, recent vaccinations, or pregnancy, and were balanced with YWH participants for sex, age and race.

Assessments were based on a single time point and included demographic characteristics, CD4 T cell counts, clinical status, antiretroviral medications, substance use, and mental health. One-way ANOVA was used to compare age, ART duration, ART regimen, and CD4 count between groups. The chi-square test was used to evaluate the influence of sex and race on HIV status. GraphPad Prism 10.0.3 (GraphPad Software, San Diego, CA, USA) was used for all statistical analysis. Statistical significance was defined as *p* < 0.05.

Substance-use assessment was performed by toxicology on plasma samples (0.5 mL) by Delta 9 Analytical LLC. (Ontario, Canada) using an LC-MS/MS assay or by Immunalysis Corporation (Pomona, CA, USA) using an ELISA. These semi-quantitative assays measured Δ9-tetrahydrocannabinol (THC), its metabolites (11-hydroxy-Δ9-THC [11-OH-THC] and 11-nor-9-carboxy-Δ9-THC [THC-COOH]), and cotinine, a nicotine metabolite. The limit of detection was 10ng/mL for the assay [8,51,52]. Substance use was defined as detectable plasma THC-COOH or cotinine at the time of blood draw. A single participant who had tested positive for methamphetamine was excluded from the analysis. Additionally, self-reported substance-use frequency over three months preceding blood collection was assessed using the World Health Organization Alcohol, Smoking, and Substance Involvement Test [53] via Audio Computer-Assisted Self-Administered Interviewing (ACASI) [8]. This captured the participants’ age at first substance use, duration, and frequency of use categorized as daily, weekly, or occasionally. Fifteen YWH included in the plasma biomarker analysis also reported weekly alcohol use, which was accounted for as a covariate in the analysis.

Multi-modal analyses were conducted using the available samples. All participants were included in plasma biomarker profiling. Due to limitations in sample quality, a nested study on a subset of samples was analyzed by flow cytometry (36 YWOH and 29 YWH) and transcriptomics (28 YWOH and 60 YWH); all these participants were included in the plasma biomarker analysis. YWH participants were further classified based on their substance-use status.

### 2.3. Plasma Biomarker Profiling, Data Processing, Normalization and Analysis

The plasma biomarker concentration of twenty-three biomarkers broadly associated with various aspects of immunity was measured using multiplex assays (Meso Scale Diagnostics, Gaithersburg, MD, USA) following the manufacturer’s protocol [54]. These included markers of macrophage activation (CRP, GM-CSF, sCD14, and sCD163), T cell activation and differentiation (sCD27, IFNγ, and IL-2RA), IFNγ-inducible chemokines (CXCL9 and CXCL10), inflammatory chemokines (CCL2, CCL4, CCL5, and IL-8), inflammatory cytokines (TNFα, IL-1β, and IL-6), anti-inflammatory or immunomodulatory cytokine (IL-10), intestinal barrier dysfunction (iFABP and LBP), neutrophil function (MPO), vascular inflammatory biomarkers (sVCAM-1 and sICAM-1), and cellular migration (MMP-2).

Biomarker concentrations (pg/mL) were log-transformed, with values below the limit of detection (LOD) assigned the minimal detectable value. All statistical analyses were performed in R/RStudio version 2024.12.0+467.

An initial exploratory analysis was conducted to assess the differences in mean log-transformed plasma biomarker concentrations across groups using univariate methods. ANOVA followed by Tukey’s post hoc test was applied when the data met the assumption of normality; otherwise, the Kruskal–Wallis test was used, followed by Dunn’s test for multiple comparisons.

For pairwise comparison, a linear mixed-effect model was used with plate as a random effect to account for batch variability. Models were adjusted for age, sex, race, alcohol use, and CD4 count. If the mixed-effect model produced a singular fit, a linear regression model with plate as a fixed effect was applied. Group differences were evaluated using ANOVA, with *p*-values adjusted for multiple testing across 23 biomarkers using the Benjamin–Hochberg correction. For comparison with the reference group, Dunnett’s adjustment was applied.

Two sets of pairwise analyses were conducted. The first compared YWH who used no substance (*n* = 37), marijuana alone (*n* = 17), tobacco alone (*n* = 10), and marijuana with tobacco (*n* = 37) to a reference group of YWOH who used no substance (*n* = 37). The second compared YWH who used marijuana (*n* = 17), tobacco (*n* = 10), and marijuana with tobacco (*n* = 37) to YWH who used no substance as a reference group (*n* = 37).

A censored linear model was used as a sensitivity analysis to assess the impact of the LOD’s effect on biomarkers with concentrations below the detection threshold. Model diagnostics included normal residual normality and residual-versus-predicted plots. All observations were initially equally weighted; outliers violating model assumptions were subsequently downweighted to 0.1, and the model was refitted. Biomarkers were examined individually to ensure analytical robustness.

### 2.4. Flow Cytometry Analysis of Lymphocyte Subsets

The absolute number and percentages of lymphocyte subpopulations were measured in whole blood using a 3-laser Cytek Auora Flow Cytometer (Cytek Bioscience, Fermont, CA, USA), as previously described [41]. Flow cytometry data were analyzed using FlowJo (version 10). Total lymphocytes were identified by CD45 gating, followed by enumeration of total CD3 T cells, CD4 T cells, CD8 T cells, CD19 B cells, and expression of activation markers CD38/HLA-DR on total CD8 T cells. Additional gating was applied to further characterize naïve (CD4, CD45RA^+^, CCR7^+^) and effector memory (CD4, CD45RO^+^, CD45RA^−^, CCR7^−^) CD4 T cells within the total CD4 population. One-way ANOVA (*p* < 0.05) was used to compare differences in the percentages of lymphocyte subpopulations across groups using GraphPad Prism 10.0.3.

### 2.5. Peripheral Blood Cell RNA Isolation, Library Preparation, Sequencing and Data Analysis

Total intracellular RNA was isolated from whole blood samples in PaxGene tubes [24,39]. RNA quality was assessed by an Agilent Bioanalyzer (Agilent Technologies, Santa Clara, CA, USA) and all samples had RNA integrity scores above 7. Globin-reduced RNA (100 ng) was used to construct the RNA-Seq library using the Poly (A) mRNA Magnetic Isolation Module and NebNext Ultra II RNA Library Prep Kit for Illumina (New England Biolabs, Ipswich, MA, USA). RNA-Seq libraries with unique barcodes were sequenced using Illumina HiSeq 3000 platform for 2 X 100 cycles (Illumina, San Diego, CA, USA) at the Interdisciplinary Center for Biotechnology Research at the University of Florida. Fifty million paired-end reads were generated for each sample.

Raw sequencing reads were checked for quality control with FASTQC version 3 [55]. The low-quality reads and residual adaptor sequences were trimmed using trimmomatic version 0.27 [56] and then aligned to the USCS reference transcriptome, which included 26,475 annotated genes [57]. Read counts for each gene were obtained, and TPM (Transcript Per Million)/FPKM (Fragments Per Kilobase Million) values were calculated. The aligned reads were also assessed for batch variation and outliers using BatchQC version 2.0.0 [58]. DESeq2/Bioconductor R package version 1.4.4.0 was used to determine the size factor for normalization, and vst variance stabilization was performed before conducting downstream analysis [59,60,61]. The sequencing data were uploaded to dbGaP [62] and will be released upon the publication of this manuscript (Accession ID phs002218.v1.p1).

### 2.6. Identification of Differentially Expressed Genes (DEGs)

Differential gene expression analysis was performed using DESeq2, correcting for batch effects and multiple testing [59,60,61]. Aligning with the plasma biomarker analysis, gene expression differences were assessed in two comparisons.

The first comparison evaluated YWH who used no substance (*n* = 27), marijuana alone (*n* = 14), and marijuana with tobacco (*n* = 19) against the reference group of YWOH who used no substance (*n* = 28). The second analysis compared YWH who used marijuana alone (*n* = 14), and marijuana with tobacco (*n* = 19) to YWH who used no substance as a reference (*n* = 27). Due to an insufficient number of samples, YWH using tobacco alone, were excluded from the gene expression analysis.

Genes with a false discovery rate (FDR) < 0.2 and (|FC|) ≥ 1.2 were selected as DEGs [63,64]. The overlap between DEGs was visualized using an UpSet plot with R package UpSet version 1.4.0 [65]. The EnhancedVolcano package version 4.5.0 in R was used to visualize the upregulated and downregulated genes of specific groups [66]. A Venn diagram was composed using the Venn Diagram R package version 1.7.3 [67]. A comprehensive list of DEGs for both the first and second comparisons of each test group, including gene name, log_2_ fold change, *p*-value, and FDR value, is provided in Table S1.

### 2.7. Functional Enrichment and Network Analysis of Differentially Expressed Genes

DEGs from each group were imported into Ingenuity Pathway Analysis (IPA) software version 1.8 (Qiagen; Bioinformatics, Redwood City, CA, USA) to identify canonical signaling pathways associated with the DEGs. IPA uses Fisher’s to calculate the *p*-value by comparing the proportion of study genes associated with a given pathway to the proportion of expected genes. To infer pathway activity, IPA calculates a z-score based on the direction of DEGs (upregulation or downregulation) relative to the expected directions of the same genes in the Ingenuity Knowledge Base [68]. Pathways with a *p* ≤ 0.001 and z-score ≥ 1 were classified as activated, while those with a *p* ≤ 0.001 and z-score ≤ −1 were classified as inhibited [69,70].

In IPA, canonical pathway names are assigned by the IPA content team based on the curated literature and experimental findings. To enhance biological interpretation and identify trends, significantly perturbed pathways were categorized by function and visualized as a network plot using the VisNetwork package version 2.1.2 [71].

## 3. Results

### 3.1. Characteristics of the Study Population Included in Bioprofiling

The YWH study population (*n* = 101) was predominantly African American (60%) and male (78%), with a median age of 24 years (Table 1). YWOH (*n* = 37) were younger (median age 22 years) than YWH (median age 24 years) but also predominantly male (68%) and African American (76%). Antiretroviral regimens included two nucleotide reverse-transcriptase inhibitors (NRTIs, emtricitabine and tenofovir) combined with either a protease inhibitor (PI, ritonavir-boosted atazanavir), a non-nucleoside reverse-transcriptase inhibitor (NNRTI, efavirenz), or an integrase strand transfer inhibitor (INSTI, raltegravir or bictegravir) for a median of 977 days. Median CD4 T cell counts (670 cells/µL) at the time of blood draw were similar across all groups and within the normal range [72]. Age, sex, days on ART, type of ART, and nadir CD4 T cell counts at the time of blood draw were similar across substance-use groups.

Substance use among the participants was defined by plasma toxicology for THC-COOH and cotinine levels. Self-reports were assessed to estimate the frequency of substance use (Figure S1). Among the 37 YWH participants with substance-negative toxicology, 71% reported never using marijuana, 84% reported never using tobacco, and the remainder reported occasional use of one or the other substance. Among the 17 participants who tested positive for marijuana, nine reported regular use (defined as either daily or weekly), seven reported occasional use (less than weekly), one reported no use, and none reported tobacco use. Among the participants who tested positive for tobacco, 70% reported daily use, while only three reported occasional or no use; none reported any marijuana use. Among the 37 YWH with positive toxicology for both marijuana and tobacco, 78% reported regular marijuana use and 62% reported regular tobacco use.

### 3.2. Marijuana Use Elevates Plasma Anti-Inflammatory Cytokine Bioprofile

To identify immune bioprofiles associated with substance use and viral suppression among YWH, log-transformed plasma biomarker concentrations were initially compared across all groups (Figure S2). Overall, YWH exhibited distinct inflammatory biomarker profiles that were further modulated by substance use.

**Figure 1:**
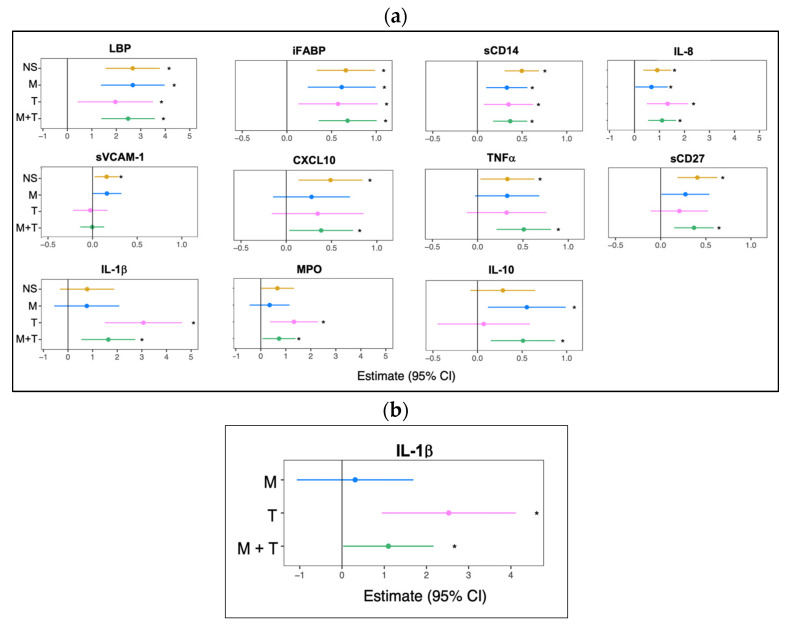
Substance use modulates distinct inflammatory biomarkers relative to youth without (**a**) and with HIV (**b**). (**a**) Forest plots showing 11 biomarkers with statistically significant differences in mean log-transformed concentrations among YWH who used no substance (NS—yellow), marijuana alone (M—blue), tobacco alone (T—pink), or marijuana with tobacco (M + T—green), compared to YWOH as the reference group. (**b**) Forest plots showing biomarkers with statistically significant differences in mean log-transformed concentrations among YWH who used marijuana (M—blue), tobacco (T—pink), or marijuana with tobacco (M + T—green), compared to YWH who used no substance (NS) as the reference group. For each panel, pairwise effect estimates and 95% confidence intervals were derived from linear regression models adjusted for age, sex, race, alcohol use, and CD4 count. Significance was evaluated using ANOVA with Benjamin–Hochberg correction for multiple testing; Dunnett’s adjustment was applied for comparison with reference groups. Significant differences in pairwise comparisons to the reference group are indicated by an asterisk (* *p* < 0.05).

Pairwise analysis compared to YWOH revealed significant differences by virus infection between YWH and YWOH in at least four immune markers, including intestinal barrier dysfunction markers LBP and iFABP, macrophage activation marker sCD14, and inflammatory chemokine IL-8 (Figure 1a). The vascular inflammatory marker sVCAM-1 was significantly elevated in the YWH no-substance use group compared to the YWOH. With substance use group, further influencing immune profiles. YWH groups using no substance and marijuana with tobacco were associated with elevated levels of interferon-inducible chemokine CXCL10, inflammatory cytokine TNF-α, or T cell activation marker sCD27 compared to YWOH, whereas tobacco use alone and marijuana with tobacco use were associated with elevated levels of the inflammatory cytokine IL-1β and neutrophil function MPO. Additionally, immune modulatory cytokine IL-10 levels were significantly elevated in YWH groups using marijuana alone or combined with tobacco, compared to YWOH.

To determine if substance use was associated with elevated immune biomarkers within YWH, a pairwise comparison was performed between the three substance-use groups and YWH who used no substance (Figure 1b). IL-1β remained significantly elevated in those using tobacco alone and marijuana with tobacco while IL-1β levels in those using marijuana alone were comparable to YWH using no substance. To determine if daily tobacco use was associated with higher plasma IL-1β levels, a sub-analysis was performed that revealed no significant differences in IL-1β (*p* = 0.57) levels between daily versus non-daily tobacco use, suggesting that the frequency of tobacco use did not significantly influence this biomarker. 

### 3.3. Lymphocyte Subpopulations Among YWH Showed Limited Modulation by Substance Use

This analysis was performed on a subset of participants included in the plasma biomarker analysis. Percentages of total CD4 and naïve CD4 T cells among YWH were comparable to those in YWOH, although co-use of marijuana and tobacco significantly increased the percentages of effector memory CD4 T cells compared to YWOH. Total CD19 B cell levels were similar among YWH, with no apparent effect of substance use relative to YWOH (Table 2). Percentages of total CD8 T cells among YWH trended higher than in YWOH.

Similarly, expression of HLA-DR and CD38 on total CD8 T cells showed no significant differences across substance-use groups. However, overall, virally suppressed YWH had higher HLA-DR and CD38 expression on CD8 T cells compared to YWOH.

### 3.4. Distinct Differential Gene Expression Profiles Associated with Substance Use in Virally Suppressed YWH

Building on plasma biomarker analyses that revealed unique immune or inflammatory bioprofiles based on substance use, transcriptome analysis was performed to obtain a more comprehensive overview of cellular landscapes, enabling inference of molecular mechanisms beyond immune modulation. The demographic and clinical characteristics of the participants included in the transcriptome analysis were a subset of those in the biomarker analysis with similar demographics (Table S2), ensuring consistency across the multiple analytical approaches.

**Figure 2:**
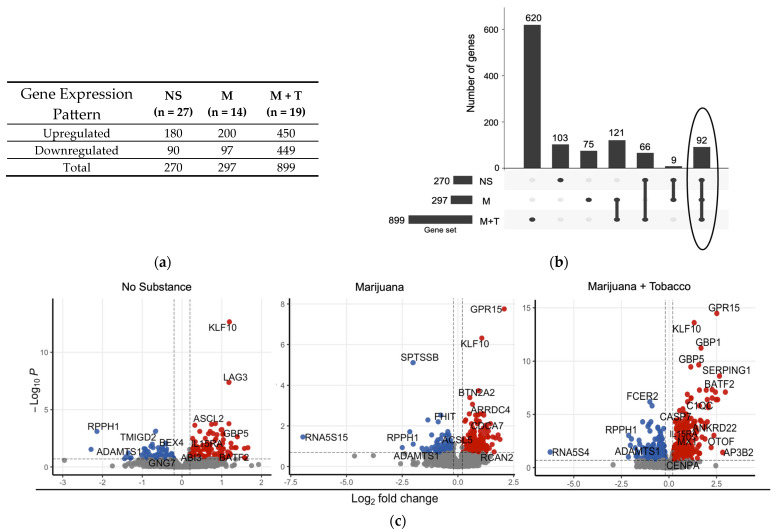
Distinct differential gene expression profiles associated with substance use in virally suppressed YWH relative to YWOH (**a**) Differential expression analysis was performed to gain a global perspective of the perturbation in gene expression by YWH and substance use using transcriptome profiles of YWOH, who used no substance as the reference. Columns show genes differentially expressed in each test group. Genes showing an absolute fold change (|FC|) ≥ 1.2 and (FDR) < 0.2 were considered significantly altered in expression. NS: YWH who used no substances; M: YWH who used marijuana; M + T: YWH who used marijuana with tobacco. (**b**) An UpSet plot was used to visualize the overlap of differentially expressed gene sets. In this vertical UpSet plot, the columns represent the individual gene sets, and the rows correspond to the intersections of these sets. Circle highlights distinct transcriptional profiles among virally suppressed YWH (**c**) A volcano plot was utilized to visualize highly significant DEGs perturbed across different groups. Here the x axis shows log_2_ FC, which represents the magnitude of change in gene expression, with positive values denoted by red dots, indicating upregulation, negative values by blue dots, denoting downregulation, and non-significant values denoted by gray dots. The y axis represents the statistical significance of changes in gene expression for different YWH substance-use groups. Only highly significant DEGs were labeled by their gene symbols.

Compared to YWOH, differential expression analysis revealed up and downregulated DEGs among YWH who used no substance, marijuana alone, or marijuana with tobacco (Figure 2a). YWH who used no substance showed dysregulation of 270 differentially expressed genes (DEGs), while marijuana alone increased the number of DEGs by about 10% to 297. Use of marijuana with tobacco increased the number of DEGs by three-fold to 899, with about half upregulated and half downregulated compared to YWOH.

The DEG overlap across the groups was visualized using an UpSet plot (Figure 2b) and a Venn diagram (Figure S3), which showed 103 DEGs (103/270, 38%) specific to the no-substance-use group, 75 DEGs (75/297, 25%) specific to marijuana use group alone, and 620 (620/899, 69%) that were exclusive to the marijuana with tobacco use group. Only 92 DEGs were shared across all groups, highlighting distinct substance-use-associated transcriptional profiles among the virally suppressed YWH.

To illustrate key DEGs, a volcano plot was used to display the distribution of highly significant DEGs among the substance-use groups (Figure 2c). Some shared DEGs, such as transcription factor *KLF10*, were upregulated in all YWH groups relative to YWOH, while shared DEGs *RPPH1* and *ADAMTS1* were consistently downregulated across all groups. Certain DEGs were more group-specific. For example, the immune check-point gene *LAG3* was upregulated in YWH who used no substance. In contrast, G protein-coupled receptor *GPR15* was similar between no-substance-use YWH and YWOH, but highly upregulated in YWH groups using marijuana alone or combined with tobacco. Guanylate-binding protein *GBP* and interleukin *IL15RA* were upregulated in the no-substance-use and marijuana-with-tobacco groups, whereas *SERPING1* and *AP3B2* were specifically upregulated in the marijuana-with-tobacco group.

Compared to YWH who used no substance, marijuana use alone only had one DEG, *GPR15*, whereas marijuana with tobacco combined use showed 47 DEGs (Table S1), with 29 upregulated and 18 downregulated. The top upregulated DEGs in the marijuana-with-tobacco group included *AP3B2*, *GPR15*, *SEMA6B*, *P2RY6*, *SERPING1*, and RSAD2. The top downregulated DEGs included *PA2G4*, *EIF2AK1*, *FIS1*, *AHSP*, and *COLQ*.

### 3.5. Canonical Pathways Related to Cellular Immune Response Normalized by Marijuana

**Figure 3:**
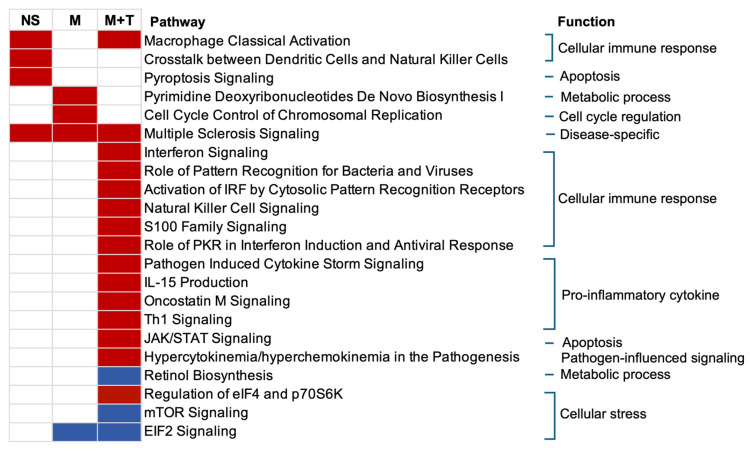
Pathway analysis reveals substance-specific perturbation in YWH groups compared to YWOH. IPA used to identify significantly perturbed pathways using *p* value < 0.001 and z-score: ≥+1 activation (red) or ≤−1 inhibition (blue). NS: YWH who used no substances; M: YWH who used marijuana alone; M + T: YWH who used marijuana with tobacco.

The inferred functional analysis of the DEGs obtained relative to YWOH (Figure 3) revealed that YWH who used no substance showed activation of three specific pathways, including macrophage classical activation signaling. This pathway was normalized by marijuana use alone but remained activated by marijuana with tobacco co-use. Other pathways perturbed in the no-substance-use group included crosstalk between dendritic cells and natural killer cells, and pyroptosis signaling. Marijuana use alone uniquely activated two pathways, namely pyrimidine deoxyribonucleotides de novo biosynthesis I and cell cycle control of chromosomal replication. In contrast, marijuana with tobacco co-use perturbed 15 pathways, including interferon signaling, pattern recognition receptors for bacteria and viruses, natural killer cell signaling, pathogen-induced cytokine storm, IL-15 production, Th1 signaling, and JAK/STAT signaling, among others. Inhibited pathways included the retinol biosynthesis pathway and mTOR signaling. EIF2 signaling was inhibited by both marijuana use alone and marijuana combined with tobacco use. The multiple sclerosis signaling pathway was shared by all groups.

**Figure 4:**
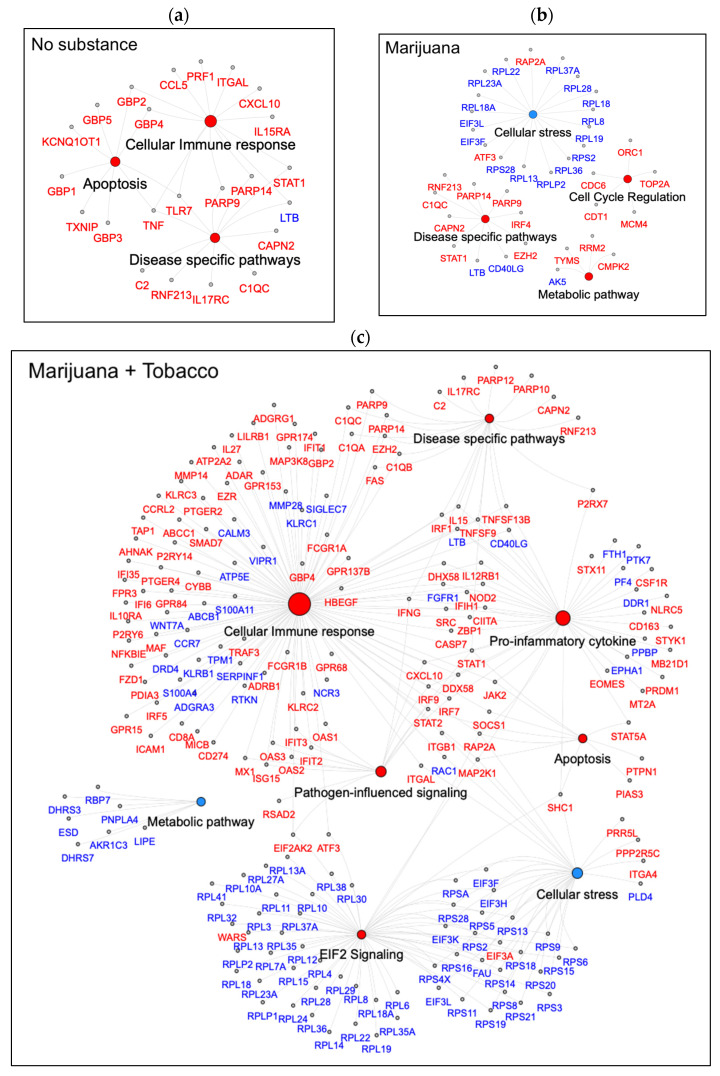
Network analysis reveals functional clustering of pathways perturbed by substance use in YWH. Network plot shows DEGs connecting the pathways for YWH using no substance ((**a**), top left panel), YWH using marijuana alone ((**b**), top right panel), and YWH using marijuana with tobacco ((**c**), bottom panel). Nodes: pathways/functions; edges: DEGs connecting the pathways. Red circles: activated pathways/function; blue: inhibited pathways/function. DEGs: red: upregulated; blue: downregulated.

Perturbed pathways were further categorized based on inferred function and visualized as network plots (Figure 4a–c). Pathways perturbed among YWH with no substance use were primarily associated with activation of programmed cell death or apoptosis and cellular immune response. Cellular immune response was normalized by marijuana alone, while activating pathways related to cell cycle regulation and metabolic process and inhibiting cellular stress. In contrast, marijuana used in combination with tobacco by YWH activated pathways linked to cellular immune response, pro-inflammatory cytokine signaling, pathogen-induced signaling, apoptosis, and regulation of eIF4. Pathways inhibited by marijuana with tobacco use were related to metabolic processes and cellular stress.

Multiple DEGs from the same families were consistently expressed in the same direction within one or more pathways across all substance-use groups. For example, poly(ADP-ribose) polymerase family genes, *PARP9* and *PARP14*, were upregulated in all groups. DEGs associated with inflammatory responses, including gene *CXCL10*, an interferon inducible chemokine, and the interleukin receptor gene *IL17RC*, in disease-specific pathways were upregulated in the no-substance-use and marijuana-with-tobacco groups. Members of the tumor necrosis factor (TNF) superfamily showed differential expression, with *TNF* specifically upregulated in the no-substance-use group, while *TNFSF13* and *TNFSF9* were uniquely upregulated in the marijuana-with-tobacco group. Guanylate-binding proteins, *GBPs*, a family of GTPase, were similarly upregulated in these two groups. The cellular stress response included downregulated genes from eukaryotic translation initiation factor 3, *EIF3* subunits, and the ribosome protein L *RPL* gene family, specifically in groups using marijuana alone and marijuana with tobacco.

The functional analysis of DEGs perturbed in YWH who co-used marijuana with tobacco (47 DEGs, Table S1) compared to YWH who used no substance showed activation of the interferon signaling pathway (cellular immune response).

## 4. Discussion

This study utilized a comprehensive multi-modal approach, including measurement of plasma immune biomarkers, analysis of peripheral blood lymphocyte subsets by flow cytometry, and transcriptome profiling of peripheral blood cells to evaluate the effects of marijuana use and/or tobacco use on immune function and molecular pathways in YWH. The findings revealed that marijuana use alone may exert an immunomodulatory effect, while tobacco use alone or combined with marijuana was associated with a heightened pro-inflammatory profile. The findings identify potential markers for early detection of inflammation-related comorbidities and emphasize the importance of targeted profiling to guide personalized monitoring in YWH as marijuana legalization expands and tobacco use persists in this population.

The study cohort primarily consisted of young African American males, a population at the highest risk of new HIV infections in the U.S. [45]. Participants were enrolled from multiple sites across the U.S., allowing for a broad representation of recreational substance-use patterns that are not limited to a single geographic region. ART was initiated early, prior to significant CD4 T cell decline, with participants remaining stable for at least 12 months on their initial ART, and none exhibiting AIDS-defining illness or comorbidities typically seen in adults with HIV [42,43,44,49]. All substance-use groups had similar demographics, treatment regimens, CD4 T cell counts and disease stages, as well as a key comparison group of YWOH balanced for sex and ethnicity.

Substance-use groups were defined based on plasma toxicology for the detection of THC-COOH and/or cotinine, with further validation of the extent of substance use using an established self-report assessment tool [53], which defined the extent of substance-use exposure as regular or occasional use. We previously showed concordance between self-reported substance use and toxicology among YWH [8]. Not surprisingly, 70 to 80% of youth who had positive cotinine toxicology, whether alone or in combination with marijuana, reported regular use. YWH with positive toxicology for THC-COOH and cotinine showed an overall heavier pattern of use, suggesting greater exposure to both substances. A small number of participants reported regular alcohol use, which was accounted for as a covariate in the statistical models for biomarker analysis. This careful classification strengthens the study, as other substance use has been suggested to confound immune and inflammatory markers [30,73,74].

Even with viral suppression, multiple immune plasma biomarkers, including biomarkers of gastrointestinal mucosal permeability, macrophage activation, and inflammation, were perturbed in YWH relative to YWOH, findings consistent with our previous reports [39,41,42]. YWH who used no substance or marijuana with tobacco were associated with elevated pro-inflammatory biomarkers CXCL10, TNFα, and sCD27, with modest attenuation of these biomarkers when marijuana or tobacco was used alone. IL-10, an immunomodulatory cytokine, was elevated in groups using marijuana alone or marijuana with tobacco. IL-10 elevation in the marijuana-with-tobacco group occurred with concurrent elevation of pro-inflammatory markers CXCL10, TNFα, and sCD27, suggesting an appropriate downregulatory effect. Tobacco use, alone or in combination with marijuana, was associated with elevated IL-1β and MPO when compared to YWOH. In addition, IL-1β was higher in YWH who used tobacco alone or with marijuana when compared to YWH who used no substance, implicating tobacco as a driver of inflammation. IL-1β is a biomarker of inflammasome activation and is implicated in the pathogenesis of chronic obstructive pulmonary disease (COPD), a frequent comorbidity in PWH who smoke cigarettes [75,76,77]. IL-1β and MPO are known biomarkers of atherosclerotic inflammation and contribute to cardiovascular disease (CVD) progression [78,79,80,81]. Elevation of these markers may reflect endothelial dysfunction due to tobacco exposure [81,82,83,84]. These findings raise the possibility of using IL-1β as a non-specific inflammatory marker of underlying immune activation, potentially aiding in the early detecting of COPD and CVD risk in YWH as they age, particularly among those with a history of tobacco use.

According to published studies, among adults with HIV, heavy, chronic use of marijuana is associated with lower levels of T cell activation based on the expression of CD38/HLA-DR [28,30], whereas tobacco smokers showed increased expression of CD38/HLA-DR on T cells compared to non-smokers [85]. Across our cohort of YWH, T cell activation was higher when compared to YWOH, indicating persistent CD8 T cell activation despite viral suppression by ART. However, HLA-DR expression on CD8 T cells did not significantly differ across substance-use groups. Furthermore, among all YWH, the proportions of total CD8, total CD4, including effector memory and naïve T cells subsets, and CD19 B cells were not significantly different. Significant attenuation of T cell activation by marijuana in PWH may only be associated with chronic, heavy, daily use, which was not typical of the substance-use patterns among YWH. Taken together, measurements of cellular activation using cell surface markers and assessment of lymphocyte subsets using flow cytometry may lack sufficient sensitivity to detect the effects of substance use among YWH.

Analyzing DEGs and perturbed canonical pathways across YWH groups provided molecular insights into the impact of viral suppression and substance use on peripheral blood cell transcriptome. While our previous study utilized microarray data [24], the current RNA-Seq approach offers enhanced resolution and dynamic range, enabling more comprehensive transcriptome profiling [86]. Given the widespread expression of cannabinoid receptors, particularly CB2, on peripheral blood immune cells including T cells, B cells, natural killer cells, dendritic cells, and macrophages, transcriptome profiling could reveal intracellular changes and regulatory mechanisms that plasma biomarkers alone may not detect in substance-use groups [87,88,89,90]. Differential expression of the CNR2 gene (which encodes for CB2 receptors) was not observed in either the group using marijuana alone or marijuana with tobacco. This may reflect the ability of cannabinoid receptor signaling to modulate immune gene expression independently of transcriptional regulation of the receptor itself [91].

The limited overlap of only 92 DEGs across all groups highlighted the distinct transcriptional profile associated with substance use. Other studies have shown that in otherwise healthy individuals, polysubstance use including marijuana, tobacco, or alcohol has minimal effects on DEGs in the peripheral blood cell transcriptome [24,92,93]. It is likely that the measurable impact of marijuana use, alone or combined with tobacco, occurs within the context of an intrinsic pro-inflammatory state, such as HIV-infection. This is reflected in the activation of cellular immune response and pro-inflammatory markers in the YWH who used no substance compared to YWOH. The disease-specific pathway of multiple sclerosis (MS) signaling was upregulated across all YWH groups, marked by shared upregulation of interferon-responsive genes such as PARP9 and PARP14, indicating persistent immune activation despite viral suppression [94]. Interleukin receptor gene IL17RC was selectively upregulated in the no-substance-use and marijuana-with-tobacco groups, indicating enhanced Th-17 mediated inflammation [95]. TNF was upregulated in no-substance-use group while TNFSF13 and TNFSF9 were uniquely upregulated in the marijuana-with-tobacco group [96], indicating that this shared pathway is differentially perturbed across substance-use groups, with distinct patterns of signaling mechanisms that may shape long-term immune and neuroinflammatory outcomes.

DEGs from the activated cellular immune response pathway were elevated in YWH who used no substance, including interferon-inducible gene *CXCL10* and interleukin receptor gene *IL15RA* [97]. These genes were normalized in those who used marijuana alone and remained upregulated in those using marijuana with tobacco, relative to YWOH. This gene expression pattern mirrors plasma biomarker data, where elevated levels of CXCL10 were also observed in the no-substance-use and marijuana-with-tobacco groups, reinforcing a sustained pro-inflammatory profile potentially exacerbated by tobacco or combined substance use.

Activation of the macrophage classical signaling pathway, part of cellular immune response, was observed in YWH who used no substance and those who used marijuana with tobacco, relative to YWOH. This pathway included upregulation of interferon-inducible GTPases, mainly *GBP2* and *GBP4*, which play a critical role in innate immune responses by promoting inflammasome assembly in macrophages [98]. *GBP2* facilitates caspase-1 activation, leading to the cleavage and secretion of pro-inflammatory cytokines IL-1β [98,99,100]. Although *IL1B* transcript levels were not significantly altered in the marijuana-with-tobacco group, plasma IL-1β levels were elevated, consistent with its post-translational regulation [98,101,102]. These findings suggest that elevated plasma IL-1β levels may reflect increased caspase-1-mediated cleavage rather than increased *IL1B* transcription. In contrast, both *GBPs’* expression and activation of macrophage signaling were attenuated in the marijuana-use-alone group, indicating a potential anti-inflammatory effect of marijuana alone. Given the established role of inflammasome signaling in the development of HIV-associated comorbidities [103,104], these transcriptome findings, together with elevated plasma IL-1β levels, underscore the clinical relevance of this inflammatory axis, particularly in the context of tobacco use. The concordance between transcriptomic and biomarker data highlights the potential for targeting inflammasome-related pathways to mitigate inflammation-driven comorbidity risk in YWH.

DEGs in EIF2 signaling, part of cellular stress response, including *EIF3* subunits and the *RPL* gene family, were not differentially expressed in YWH using no substance but were consistently downregulated in those using marijuana alone and marijuana with tobacco compared to YWOH. EIF2 signaling is induced in HIV infection and can persist with ART, contributing to ongoing cellular stress and immune activation [105,106]. The inhibition of this pathway in marijuana using groups suggests a marijuana-specific effect that suppresses protein translation and stress responses.

The G protein-coupled receptor *GPR15* was specifically upregulated in both marijuana-using groups relative to YWOH and YWH using no substance. *GPR15* expression is regulated through DNA methylation and is considered a peripheral marker of epigenetic changes associated with cannabis and tobacco smoke exposure in primarily African American young adults [107,108,109]. The consistent upregulation of *GPR15* may reflect marijuana-associated epigenetic modulation in the context of HIV suppression. This study is the first to demonstrate *GPR15* upregulation in HIV-positive youth using marijuana and to describe potential marijuana-associated epigenetic modulation in this population. *GPR15* is a co-receptor for HIV, especially HIV-2 and SIV [110,111], and a known homing receptor that plays a role in the T cell migration of T regulatory cells to the colon [112]. Increased *GPR15* expression in both marijuana-using groups may indicate enhanced gut-homing potential of CD4 T cells, potentially aiding in the restoration of mucosal immunity disrupted by HIV [113].

Activation of cell cycle regulation pathways, including cell cycle control of chromosome replication as well as the metabolic pathway involved in pyrimidine deoxyribonucleotides de novo synthesis I, was observed in the marijuana-alone group. Cell cycle regulation pathways included genes involved in DNA replication and cell proliferation (*ORC1*, *TOP2A*, *CDC6*, and *MCM4*) [114,115], while metabolic pathways are characterized by gene crucial for nucleotide metabolism and DNA synthesis (*RRM2*, *TYMS*, and *CMPK2*) [116]. These findings suggest potential alterations in cellular metabolism and proliferation, which may reflect increased DNA repair or replication associated with marijuana. Overall, marijuana used alone was associated with a normalizing effect on genes involved in immune activation and downstream immune signaling pathways. However, this immune modulating effect was lost when marijuana was used with tobacco, which instead induced a heightened pro-inflammatory transcriptomic profile.

The findings of this study provide insights into plasma biomarker profiles and transcriptional alteration associated with substance use in YWH. However, direct correlations between RNA expression and plasma biomarker levels remain complex to interpret due to post-transcriptional and post-translational regulatory mechanism, such as mRNA stability, translational efficiency, protein turnover, and proteolytic shedding [39]. For example, although *IL1B* transcript levels were not significantly altered in the M + T group, elevated plasma IL-1β levels were observed, which are regulated at the post-translational level [102]. Future integrative analyses that combine transcriptomic and proteomic data will be important to fully elucidate these relationships.

The YWH in this study were on ART regimens that aligned with the standard of care at the time and were consistent across groups to minimize potential ART-related effects on the bioprofiles [24,39]. The findings offer valuable insight into real-world patterns of recreational marijuana use alone or in combination with tobacco among virally suppressed YWH. This is particularly relevant given the growing public health interest in putative anti-inflammatory effects of marijuana. Medical marijuana and its derivatives are currently used by PWH to manage symptoms such as pain, weight loss, and nausea [117,118,119]. Dronabinol, a synthetic form of THC, is FDA-approved for managing HIV-associated anorexia [120,121]. Although this study was not designed as a controlled clinical trial to evaluate the effect of marijuana use, the mode of use and level of exposure to marijuana and tobacco were similar within the cohort. The specific effect of cumulative exposure, additional marijuana metabolites, or combustion byproducts were beyond the scope of this analysis. Moreover, flow cytometry and transcriptome analyses were performed only on a subset of participants due to sample availability, which may limit the statistical power of the results. Controlled clinical trials using self-administered or prescribed marijuana or isolated cannabinoids such as THC or CBD are reported [31,90,122]. By employing a multi-layered approach of biomarker profiling, flow cytometry, and transcriptomic analysis, this study offers a broader perspective on potential immunological and molecular effects of marijuana and/or tobacco use in YWH. Such multi-modal assessment can capture distinct aspects of immune function and may help identify early indicators of dysregulation that single-method studies could miss.

## 5. Conclusions

YWH demonstrated persistent immune activation even after sustained viral suppression, which is further differentially influenced by substance use. Marijuana use alone was associated with elevated plasma IL-10 levels and normalized inflammation-related genes and pathways, suggesting a potential anti-inflammatory or immunoregulatory role. In contrast, tobacco use alone or combined with marijuana was associated with a pronounced pro-inflammatory profile, marked by elevated plasma IL-1β levels. Marijuana combined with tobacco use also led to upregulation of *GBP* genes involved in inflammasome activation. These findings support the potential use of IL-1β as a marker for early detection of inflammation-related comorbidities such as COPD and CVD. This study is the first to demonstrate *GPR15* upregulation and potential marijuana-associated epigenetic modulation in HIV suppressed youth. By utilizing biomarker and transcriptomic data, the study underscores the value of a multi-modal approach to detect early immune alterations. The findings of this study have broader public health implications, supporting the use of targeted immune markers to guide personalized monitoring and early intervention strategies for youth with HIV, particularly as marijuana legalization expands and tobacco use persists in this population.

## Figures and Tables

**Table 1:** Demographics and clinical characteristics of study participants (*N* = 138).

Characteristics		Youth Without HIV	Youth with HIV (VL ≤ 50)				*p*-Value
		(*n* = 37)	(*n* = 37)	(*n* = 17)	(*n* = 10)	(*n* = 37)	
Substance use (toxicology)		No	No	M	T	M + T	
Age(years) ^a^		22 [20, 23]	24 [23, 26] *	24 [22, 25] *	24 [23, 25] *	24 [23, 25] *	<0.0001 *
Male (%)		68	84	87	70	86	0.08
African American (%)		76	55	65	40	59	0.70
Days on ART ^a^		NA	980 [586, 1058]	707 [358, 1018]	1000 [736, 1056]	973 [909, 1054]	0.43
ART regimen (%) ^b^	NRTI	NA	86	89	67	84	0.72
	NNRTI	NA	31	17	33	38	
	PI	NA	47	44	56	49	
	INSTI	NA	11	17	11	8	
CD4 T cells (number/µL) ^a,c^		751 [495, 885]	670 [555, 919]	645 [508, 818]	756 [710, 920]	666 [438, 837]	0.91
CD4 T cells nadir (number/µL) ^a^		-	451 [341, 490]	625 [521, 629]	508 [445, 561]	395 [295, 486]	0.18

^a^ Median [25th and 75th quartile range]. ^b^ Percent of youth receiving respective classes of ART. M: marijuana; T: tobacco. NA: not applicable. ^c^ CD4 at the time of blood draw. NRTIs: nucleoside reverse-transcriptase inhibitors. NNRTIs: non-nucleoside reverse-transcriptase inhibitors. PIs: protease inhibitors. INSTI: integrase strand transfer inhibitor. VL: viral load. * YWOH group was younger than YWH no substance (*p* = <0.0001), marijuana alone (<0.0001), tobacco alone (0.003), or marijuana + tobacco (0.003) using one-way ANOVA. Sex and race did not predict HIV status using chi-square test. ART duration, regimen, and nadir CD4 T cells were comparable across all YWH groups. Statistical significance defined as *p* value < 0.05.

**Table 2:** Percentages of lymphocyte subpopulation.

Percent of Cells at End of the Study ^a^	Youth Without HIV	Youth with HIV (VL ≤ 50)			
		NS	M	T	M + T
Total CD4 cells	45.9 [35.4, 53.7]	42.6 [38.3, 48.2]	44.6 [42.3, 48.1]	35.7 [34.4, 44.7]	42.9 [38.0, 45.9]
Naive CD4 T cells	41.4 [36.3, 56.8]	41.1 [38.5, 47.6]	47.7 [35.4, 55.2]	49.5 [46.0, 56.0]	45.3 [38.6, 52.4]
Effector Memory CD4 T cells	9.2 [7.1, 11.0]	13.9 [9.7, 16.5]	11.6 [10.7, 15.5]	13.3 [10.7, 15.6]	17.7 [15.8, 19.1] *
Total CD8 T cells	24.4 [20.5, 28.7]	32.3 [28.1, 36.7] *	35.1 [34.3, 35.2] *	30.8 [26.8, 33.2]	32.4 [27.8, 37.1] *
HLA-DR, CD38 CD8 T cells	0.3 [0.2, 0.5]	1.1 [0.5, 1.9]	0.5 [0.4, 1.0]	0.5 [0.4, 0.6]	0.6 [0.4, 1.5] *
Total CD19 B cells	12.7 [9.5, 17.3]	10.1 [6.8, 11.7]	11.2 [7.4, 18.4]	12.7 [10.3, 14.8]	11.9 [8.3, 16.9]

^a^ Median [25th and 75th quartile range]. * YWH substance-use groups that were significantly different from YWOH based on one-way ANOVA (*p* < 0.05).

## Data Availability

The data were uploaded to dbGaP [62] and will be released upon the publication of this manuscript (Accession ID phs002218.v1.p1).

## Supplementary Materials

The following supporting information can be downloaded at: https://www.mdpi.com/article/10.3390/cells14161267/s1, Figure S1: Concordance between toxicology and self-report substance use; Figure S2: Comparison of plasma biomarker concentrations across the study groups; Figure S3: Venn diagram showing gene overlap; Table S1: List of DEGs across both comparisons; Table S2: Demographics and clinical characteristics of study participants included in transcriptome analysis.
