# Supplementary material for: Multi-Modal Profiling Reveals Contrasting Immunomodulatory Effects of Recreational Marijuana Used Alone or with Tobacco in Youth with HIV
Source: Cells. 2025 Aug 16;14(16):1267. doi: 10.3390/cells14161267 (PMC12385114; doi:10.3390/cells14161267)
Supplement: Supplementary file 1 [file cells-14-01267-s001.zip › nida_cell_format_supp_figure_06262026.pdf]

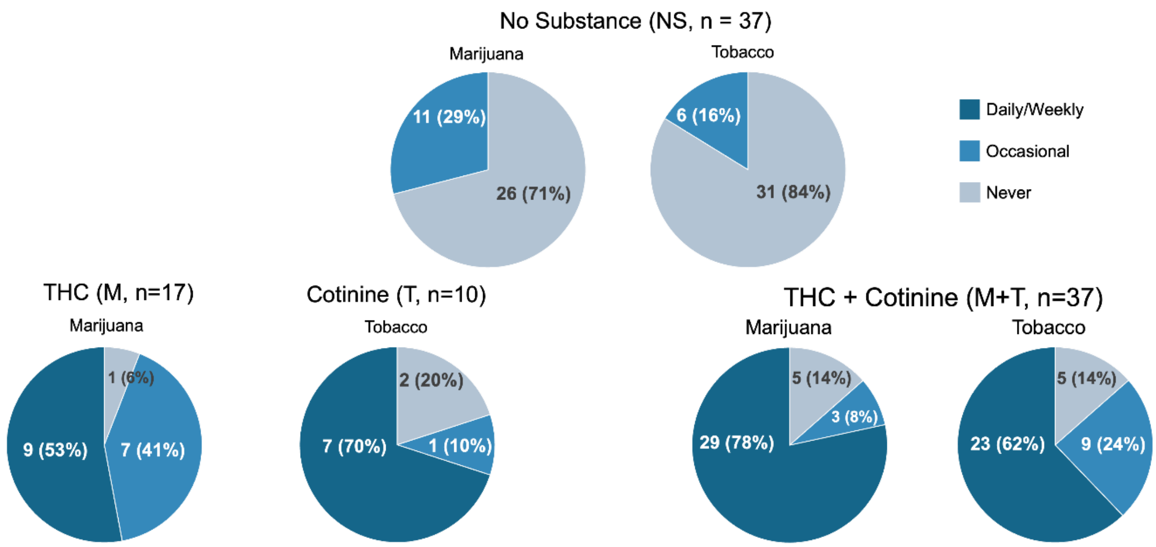

Figure S1. Concordance between toxicology results and self-reported substance use. Pie charts illustrate the frequency of self-reported tobacco and marijuana use among participants grouped by toxicology results: negative, THC only, cotinine only, or both THC and cotinine positive. Pie charts represent the number and percentage of participants self-reports classified as daily/weekly, occasional, or never using tobacco or marijuana.

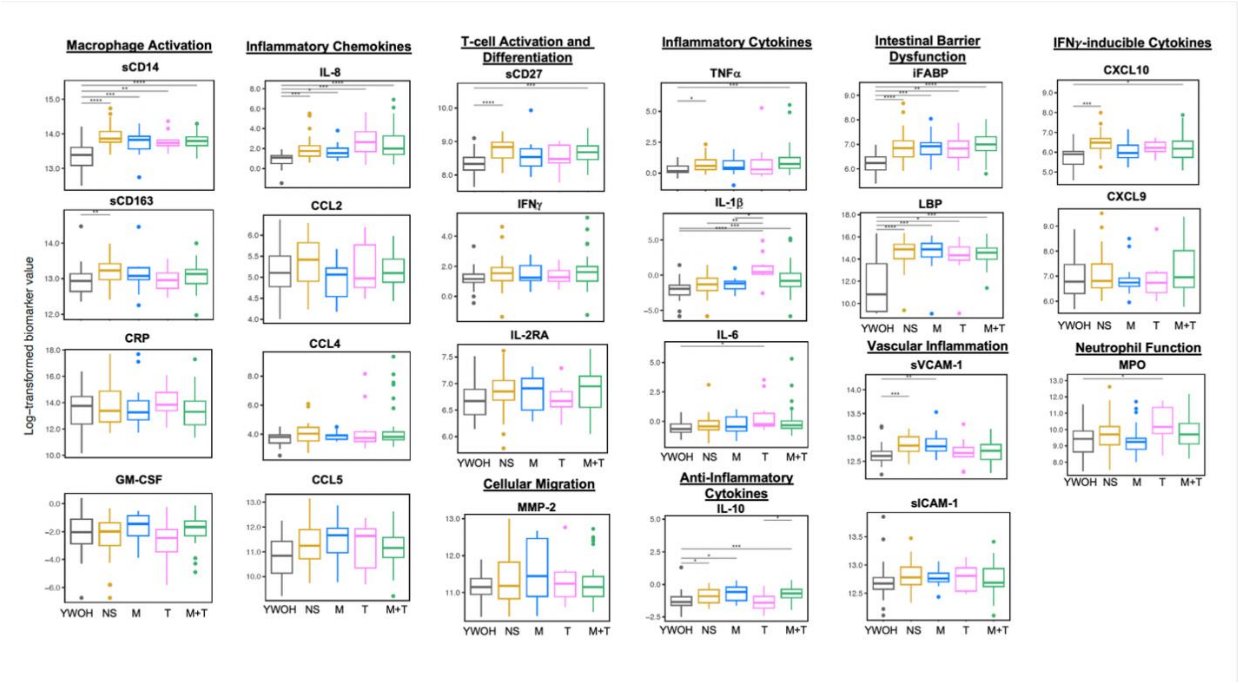

Figure S2. Comparison of plasma biomarker concentrations across study groups. Boxplots showing plasma biomarker concentrations across all study groups. Group differences were assessed using ANOVA, with p-values adjusted for multiple testing across 23 biomarkers using the Benjamini–Hochberg correction ($p < 0.05$). Biomarkers are grouped based on immune function, and the y-axis represents log-transformed biomarker concentrations (pg/mL). Boxplot colors indicate study groups: Grey – YWOH who used no substances, Yellow – YWH who used no substances (NS), Blue – YWH who used marijuana (M), Pink – YWH who used tobacco (T), Green – YWH who used marijuana with tobacco (M+T). Significant differences in group comparisons are indicated by an asterisk (* $p < 0.05$).

Table S1. List of DEGs across all comparison (submitted separately as an Excel file).

Table S2. Demographics and clinical characteristics of study participants included in transcriptome analysis (n = 88)

Characteristics	Youth without HIV (YWOH)	Youth with HIV (VL ≤ 50)			P value
	(n = 28)	(n = 27)	(n = 14)	(n = 19)	
**Substance use**	No	No	M	M+T	
**Age (years)**[a]	22 [20, 23]	24 [24, 26]*	24[22, 25]*	24 [24, 25]*	<0.0001*
**Male (%)**	64	81	79	95	0.08
**African American (%)**	64	59	64	58	0.70
**Days on ART**	NA	980 [482, 1054]	518 [325, 963]	935 [750, 975]	0.24
**CD4 T-cells (number/μL)**[a]	783 [586, 959]	658 [567, 840]	592[522, 766]	672 [433, 856]	0.90
**Nadir CD4 T-cells (number/μL)**[a]	-	418 [322, 476]	625 [520, 628]	395 [288, 495]	0.36

[a] Median [25th and 75th quartile range M: Marijuana, T: Tobacco. VL: viral load. *YWOH group was younger than YWH no substance (p = <0.0001), marijuana alone (0.024), or marijuana + tobacco (0.0009) using one-way ANOVA. Sex and race did not predict HIV status using chi-square test. Median CD4 T cell count was similar across all groups. ART duration and nadir CD4 T cells were comparable across all YWH groups based on One-way Anova. Statistical significance defined as *p* value < 0.05.

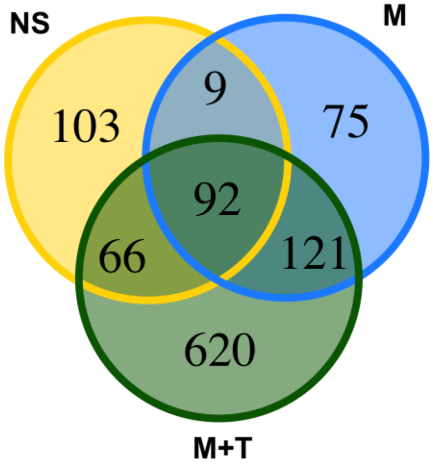

Figure S3. Venn diagram showing gene overlap. Differentially expressed genes (DEGs) from the comparisons of YWH who used no substance (NS), marijuana alone (M), and marijuana with tobacco (M+T) relative to YWOH who used no substance were plotted as a Venn diagram to illustrate gene overlap among the groups.
