# Supplementary figures and images for: Multi-Modal Profiling Reveals Contrasting Immunomodulatory Effects of Recreational Marijuana Used Alone or with Tobacco in Youth with HIV
Source: Cells. 2025 Aug 16;14(16):1267. doi: 10.3390/cells14161267 (PMC12385114; doi:10.3390/cells14161267)

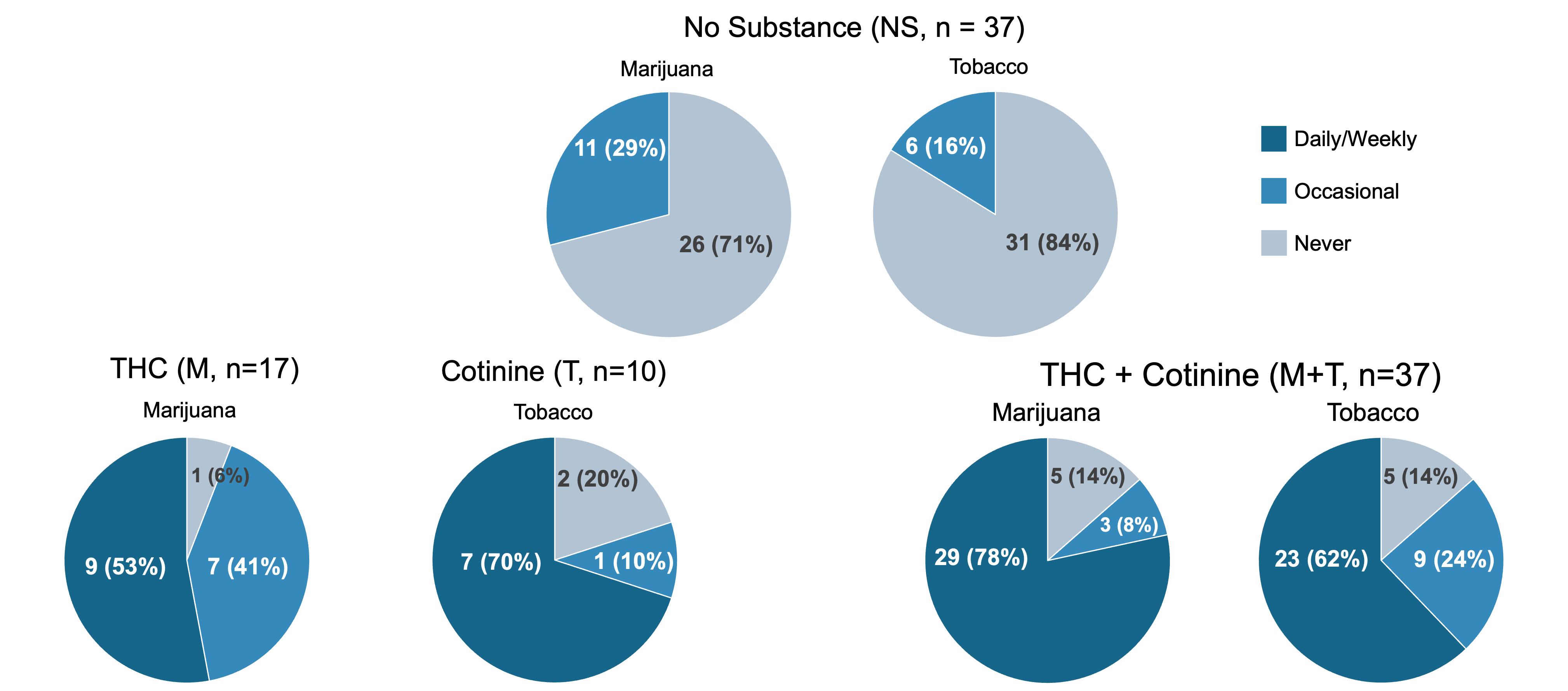

Supplement: Supplementary file 1 [file cells-14-01267-s001.zip › FigS1.png]

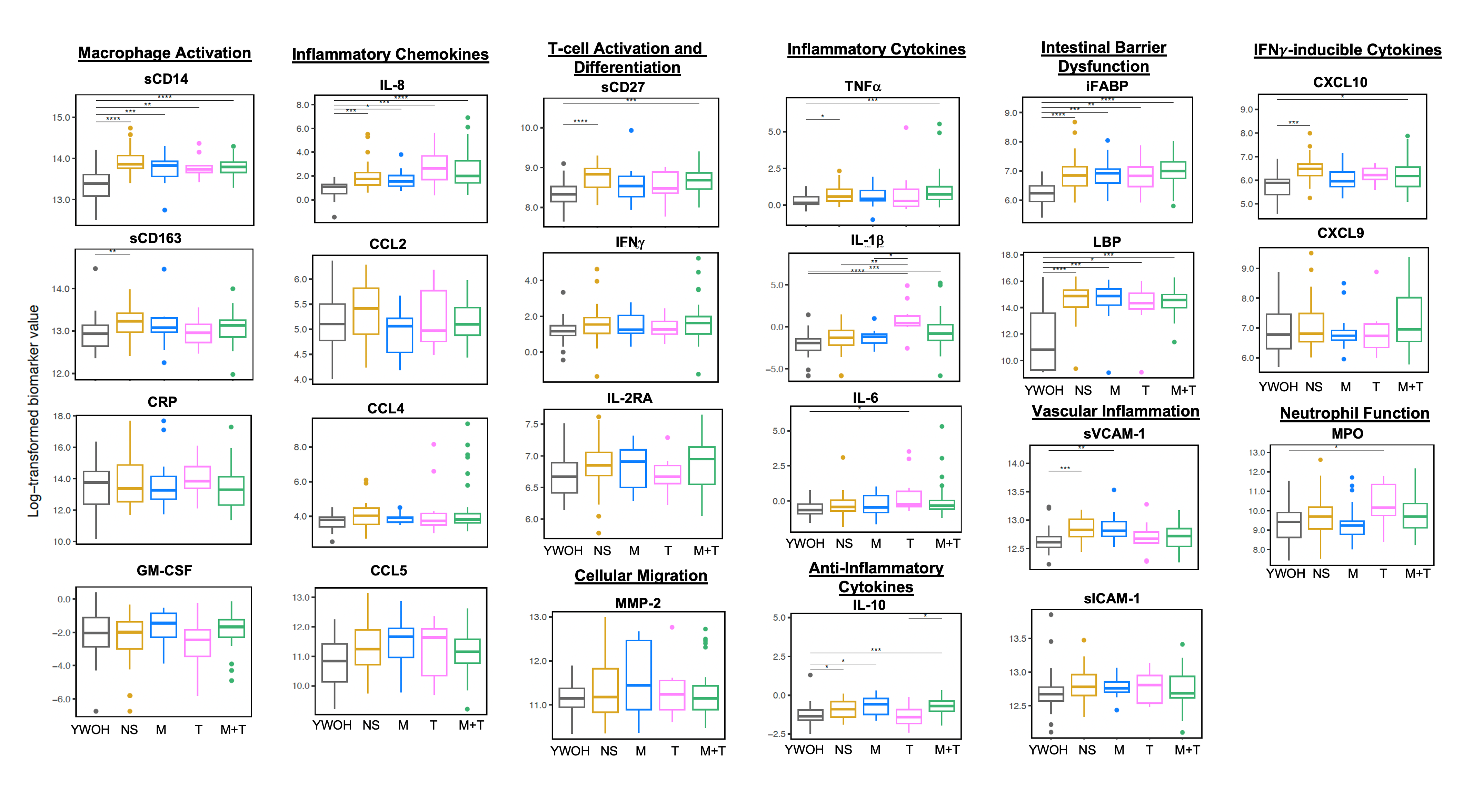

Supplement: Supplementary file 1 [file cells-14-01267-s001.zip › FigS2.png]

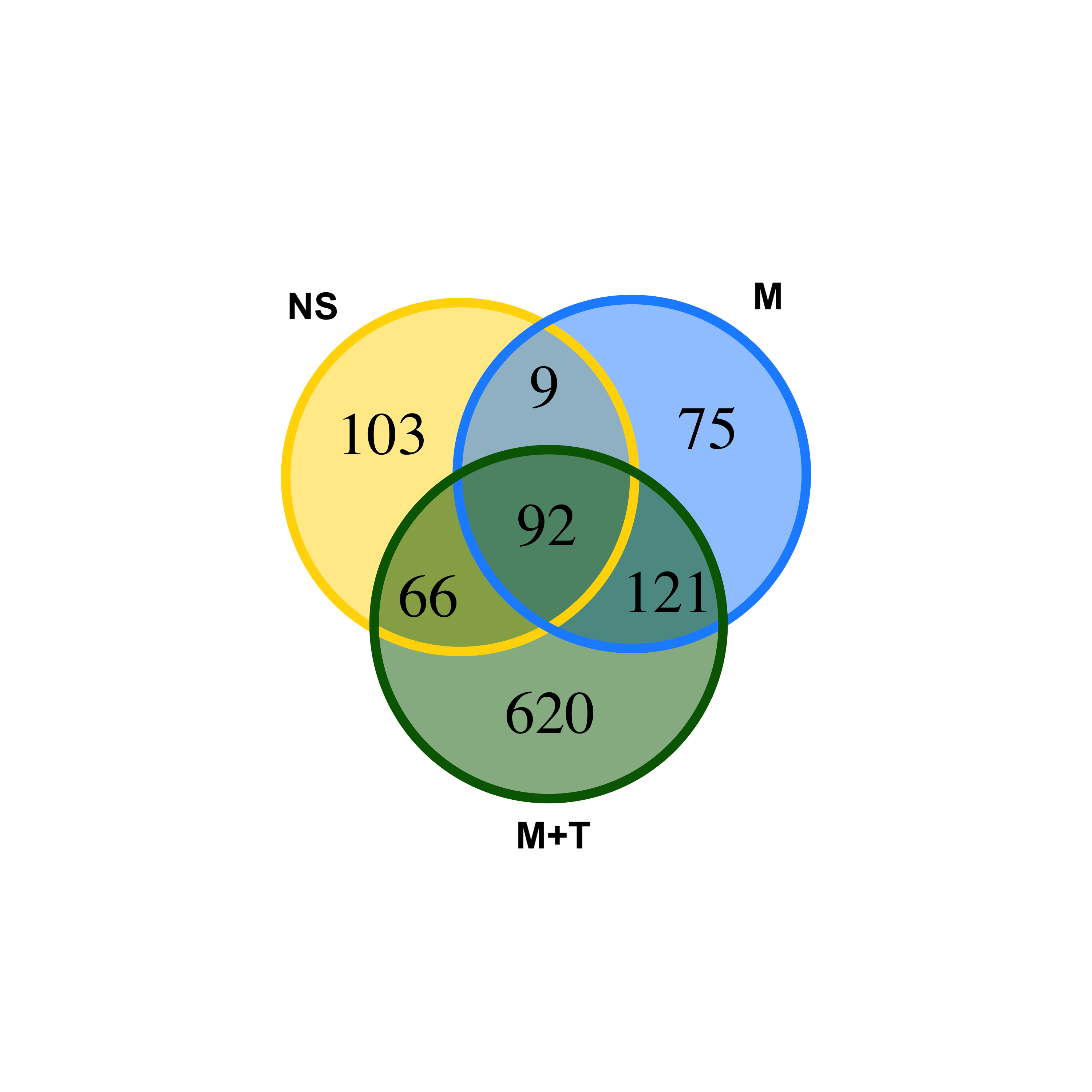

Supplement: Supplementary file 1 [file cells-14-01267-s001.zip › FigS3.tiff]
